# A New Murine Model for Gastrointestinal Anthrax Infection

**DOI:** 10.1371/journal.pone.0066943

**Published:** 2013-06-18

**Authors:** Tao Xie, Chen Sun, Kadriye Uslu, Roger D. Auth, Hui Fang, Weiming Ouyang, David M. Frucht

**Affiliations:** Laboratory of Cell Biology, Division of Monoclonal Antibodies, Office of Biotechnology Products, Center for Drug Evaluation and Research, United States Food and Drug Administration, Bethesda, Maryland, United States of America; Charité-University Medicine Berlin, Germany

## Abstract

The scientific community has been restricted by the lack of a practical and informative animal model of gastrointestinal infection with vegetative *Bacillus anthracis*. We herein report the development of a murine model of gastrointestinal anthrax infection by gavage of vegetative Sterne strain of *Bacillus anthracis* into the complement-deficient A/J mouse strain. Mice infected in this manner developed lethal infections in a dose-dependent manner and died 30 h-5 d following gavage. Histological findings were consistent with penetration and growth of the bacilli within the intestinal villi, with subsequent dissemination into major organs including the spleen, liver, kidney and lung. Blood cultures confirmed anthrax bacteremia in all moribund animals, with approximately 1/3 showing co-infection with commensal enteric organisms. However, no evidence of immune activation was observed during infection. Time-course experiments revealed early compromise of the intestinal epithelium, characterized by villus blunting and ulceration in the ileum and jejunum. A decrease in body temperature was most predictive of near-term lethality. Antibiotic treatment of infected animals 24 h following high-dose bacterial gavage protected all animals, demonstrating the utility of this animal model in evaluating potential therapeutics.

## Introduction

Recent bioterrorism attacks [1] have focused research on the inhalational route of entry, yet there remains scientific utility in investigating pathogenic mechanisms involved in gastrointestinal anthrax, as it is widely held that it is primarily the enteric route of entry that *Bacillus anthracis* has evolved to exploit [2], [3]. *Bacillus anthracis* infection is naturally acquired by ruminant herbivores that are exposed to spores when feeding in contaminated fields [2], [3]. Ruminants are considered to be the most susceptible group within the mammalian class [3]. However, it has not yet been established when and where spore germination occurs following oral consumption [3]. We have previously shown that anthrax lethal toxin (LT), which is produced by vegetative *Bacillus anthracis*, elicits rapid breakdown in the gastrointestinal barrier, characterized by villus blunting, hemorrhage, and ulceration [4], [5]. We have since been eager to develop an informative animal model that could address the role of anthrax LT, as well as other virulence factors, in mediating host-pathogen interactions during gastrointestinal infection *in vivo*. Unfortunately, the scientific community has lacked a murine model of gastrointestinal infection that incorporates administration of vegetative *Bacillus anthracis*, which could be used to investigate pathogenicity via this mode of transmission.

Previously reported animal models for anthrax infection have mainly involved the administration of *B. anthracis* spores via inhalational or parenteral routes [6], [7], [8], [9], [10], [11], [12]. Initial studies with anthrax spores administered via the gastrointestinal route failed to establish anthrax infection models in various animal species [13], [14], [15]. However, there have been recent reports of the establishment of infections in mice receiving intragastric *B. anthracis* spores [16], [17]. One group administered 10^8^ spores of an encapsulated non-toxigenic strain and reported that *B. anthracis* expanded in the Peyer’s patches, eventually disseminating into various organs. However, this model was not capable of assessing the roles of LT in promoting virulence during gastrointestinal infection. Very recently, another model was reported that utilized intragastric administration of spores embedded in a thiobendazole paste [17]. Neither of these models assessed administration of vegetative bacteria.

As ruminant animals use bacterial fermentation to facilitate digestion, we considered the possibility that in the setting of natural gastrointestinal infection, the upper gastrointestinal tract would be exposed to large numbers of vegetative bacteria. *Bacillus* species have been shown to germinate and thrive in the conditions present in the rumen [18]. For this reason, it would seem very likely that *B. anthracis* spores would germinate and proliferate in the rumen of infected animals prior to establishing infection. Under this scenario, exposure of the gastrointestinal barrier to vegetative bacteria and the toxins they produce would then lead to barrier penetration and subsequent dissemination.

We herein report that we have modeled this scenario in A/J mice through gavage of vegetative bacteria from the *Bacillus anthracis* Sterne strain. Mice infected with toxigenic bacteria via this route develop gastrointestinal disease, which leads to bacteremia and lethal dissemination. Moreover, we demonstrate that this animal model can be used to assess the efficacy of potential therapeutics.

## Results

### Intragastric Administration of Vegetative *B. anthracis* Sterne Strain (BaS) Results in Systemic Anthrax Infection

**Figure 1:**
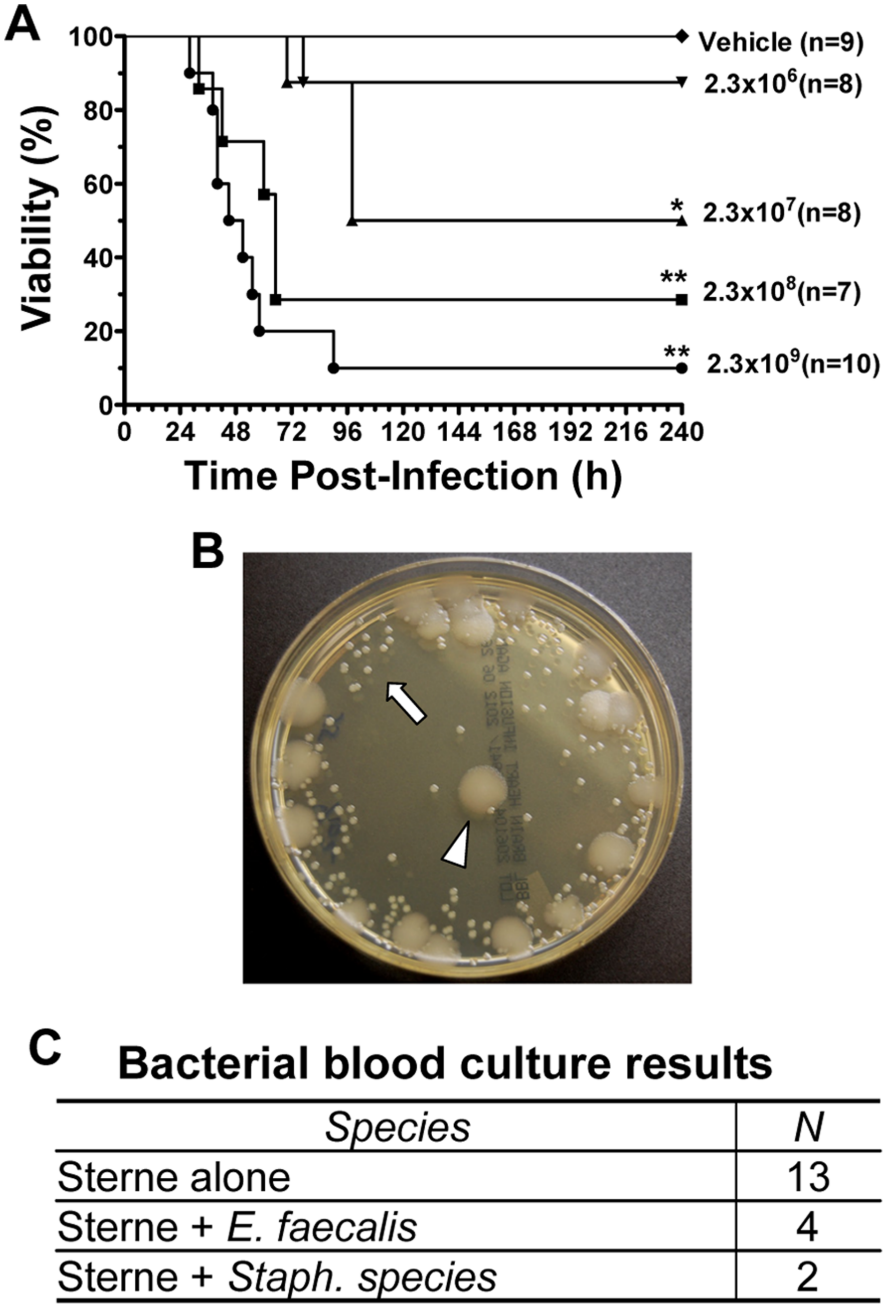
Intragastric challenge of *B anthracis* Sterne strain causes lethal anthrax infection. (**A**) Cohorts of ten week old female A/J mice were gavaged with varying doses of vegetative bacteria or with the PBS vehicle alone as indicated. The percentage of surviving/non-moribund mice was assessed at 6 h intervals for a total of 10 d as shown [p values were calculated using the log-rank (Mantel-Cox) test, * P<0.05, **P<0.01]. (**B**) A photographic image was taken of a BHI agar culture of blood from a representative animal that developed a co-infection following BaS gavage. The arrowhead indicates a bacterial colony of BaS, whereas the arrow points colonies of *Enterococcus faecalis*. (**C**) This table summarizes blood culture and bacterial identification results from 19 moribund animals.

We hypothesized that we could establish gastrointestinal infections in mice by infecting mice with vegetative bacteria, thereby mimicking conditions that we consider likely to be present during digestion in ruminants, the predominant hosts of infection in nature. To investigate this possibility, we administered increasing concentrations of vegetative *Bacillus anthracis* Sterne strain (BaS) bacteria via gavage. The percentage of mice that succumbed to this treatment increased in a dose-dependent manner (**Figure 1A** **)**. At the highest dose (2.3×10^9^), 9 of 10 mice died within 4 days of administration. In contrast, mice that received the vehicle alone showed no signs of toxicity and were blood culture negative. The LD_50_ for infection via this route in this experiment was approximately 2.3×10^7^ bacteria. One animal succumbed to infection with the lowest administered dose of 2.3×10^6^ bacteria. Interestingly 6/19 mice that were blood culture positive for BaS had mixed infections with commensal bacteria; 4/19 were blood culture positive for *Enterococcus faecalis*, whereas 2/19 were blood culture positive for enteric *Staphylococcus* species (**Figures 1B and 1C**). All mice that died in response to intragastric challenge were confirmed to be blood culture positive for BaS. To confirm that infections were due to vegetative bacteria, and not heat-resistant spores, mice were gavaged with 2×10^8^ heat-treated bacteria. None of these mice (0/5) became infected, indicating that bacterial spores were not responsible for gastrointestinal infections in our gavage model.

### Murine Gastrointestinal Anthrax has Characteristic Clinical Features

**Figure 2:**
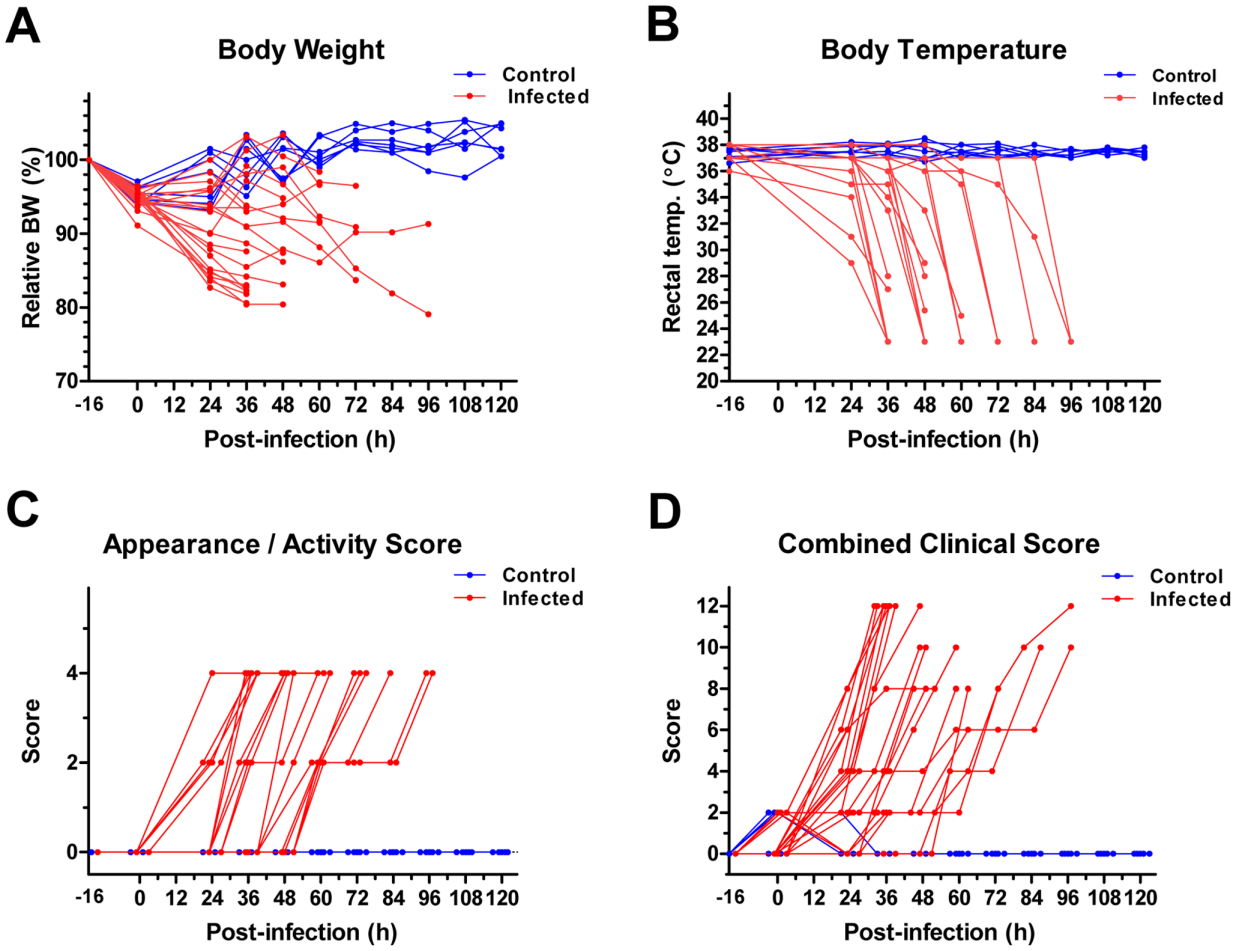
Gastrointestinal anthrax has time-dependent effects on various clinical parameters. Mice were fasted for 16 h and subsequently treated with ∼10^9^ intragastric BaS or PBS control. Body weight (BW), body temperature (BT), and appearance/activity were assessed prior to the fasting period and were subsequently monitored every 12 h until the animals became moribund, died, or reached the termination of the experiment (120 h). BW values for each animal were normalized to their baseline weights, which were assigned the value of 100% (**A**). BTs for each animal were assessed via a digital rectal thermometer (**B**). Appearance/activity for each animal was scored as follows: no change: (0 points); decreased locomotion, ruffled fur (2 points); and isolation, minimal spontaneous locomotion (4 points) (**C**). Combined clinical scores for each animal are shown in **D**. These scores were calculated from the 3 parameters described above. BW scores were assigned as follows: <5% change (0 points), ≥5%, but <10% (2 points), and ≥10% (4 points). BT scores were scored as follows: rectal temperature ≥35°C (0 points), body temperature <35°C, but ≥30°C (2 points), and body temperature <30°C (4 points). These scores were added to the appearance/activity score to provide the combined clinical score, with 12 representing the maximal possible clinical score.

We next characterized the clinical features of mice infected with anthrax via the gastrointestinal route. As shown in **Figure 2A**, a decline in body weight was noted following the 16 h fast that preceded the gavage. Thereafter, body weights in animals gavaged with *Bacillus anthracis* generally continued a slow trend downward, whereas the body weights of those receiving the vehicle alone returned to baseline levels. In contrast, a sudden drop in body temperature (**Figure 2B**) predicted mortality in anthrax-infected animals within 12–24 h. Similar to the decrease in body weight, a decline in appearance and activity was also noted in anthrax-infected animals as soon as 24 h post gavage, yet the time until death following the decrease in appearance/activity was variable, sometimes exceeding 24 h (**Figure 2C**). A combined clinical score that included these body weight, body temperature, and appearance/activity distinguished infected mice from non-infected controls starting 24 h post gavage (**Figure 2D**).

### Murine Gastrointestinal Anthrax is Associated with Gross Intestinal Hemorrhage and Edema

**Figure 3:**
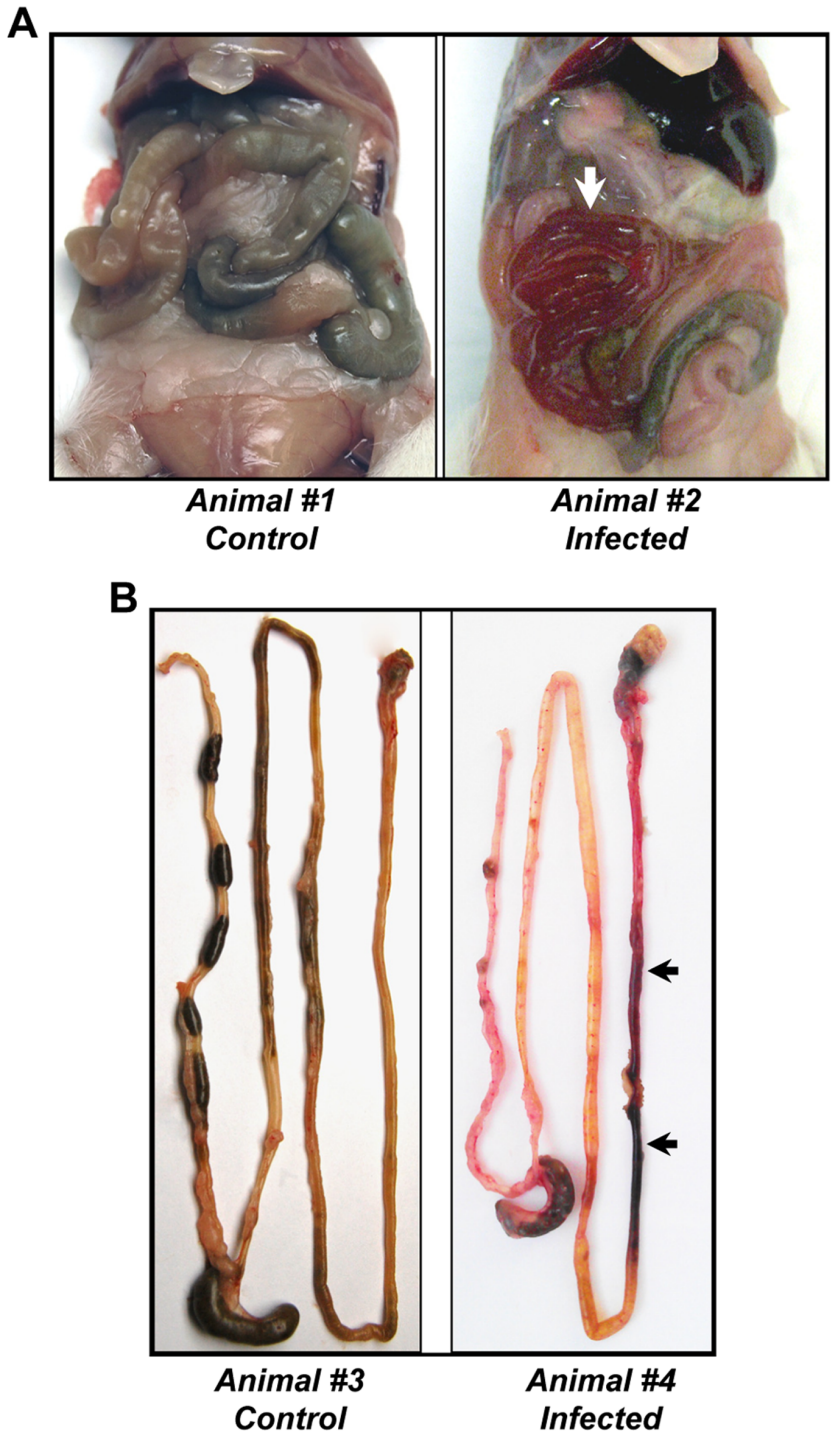
Gastrointestinal anthrax infection in mice is characterized by gross hemorrhage. Mice that were treated with intragastric BaS were euthanized when they became moribund, simultaneously with control mice administered the buffer control. Shown are representative gross autopsy findings noted upon exposure of the abdominal cavity (**A**) or following dissection of the gastrointestinal tract (**B**). Control animals are shown in the left panels, and BaS infected animals are shown in the right panels. Arrows indicate regions with blood evident within the intestinal lumen.

Mice that became moribund following development of anthrax infection were euthanized and autopsied. In contrast to control animals that received intragastric administration of vehicle alone (**Figures 3A and 3B**, left panels), mice that received BaS showed evidence of intestinal hemorrhage (**Figures 3A and 3B**, right panels). These pathological findings of gross hemorrhage were similar to those that we have reported in mice treated with anthrax LT alone [4], [5]. In addition, infected mice showed evidence of edema within the peritoneal cavity (**Figure 3A**, right panel).

### Anthrax Infection Causes Breakdown of the Gastrointestinal Barrier and Local Bacterial Invasion

**Figure 4:**
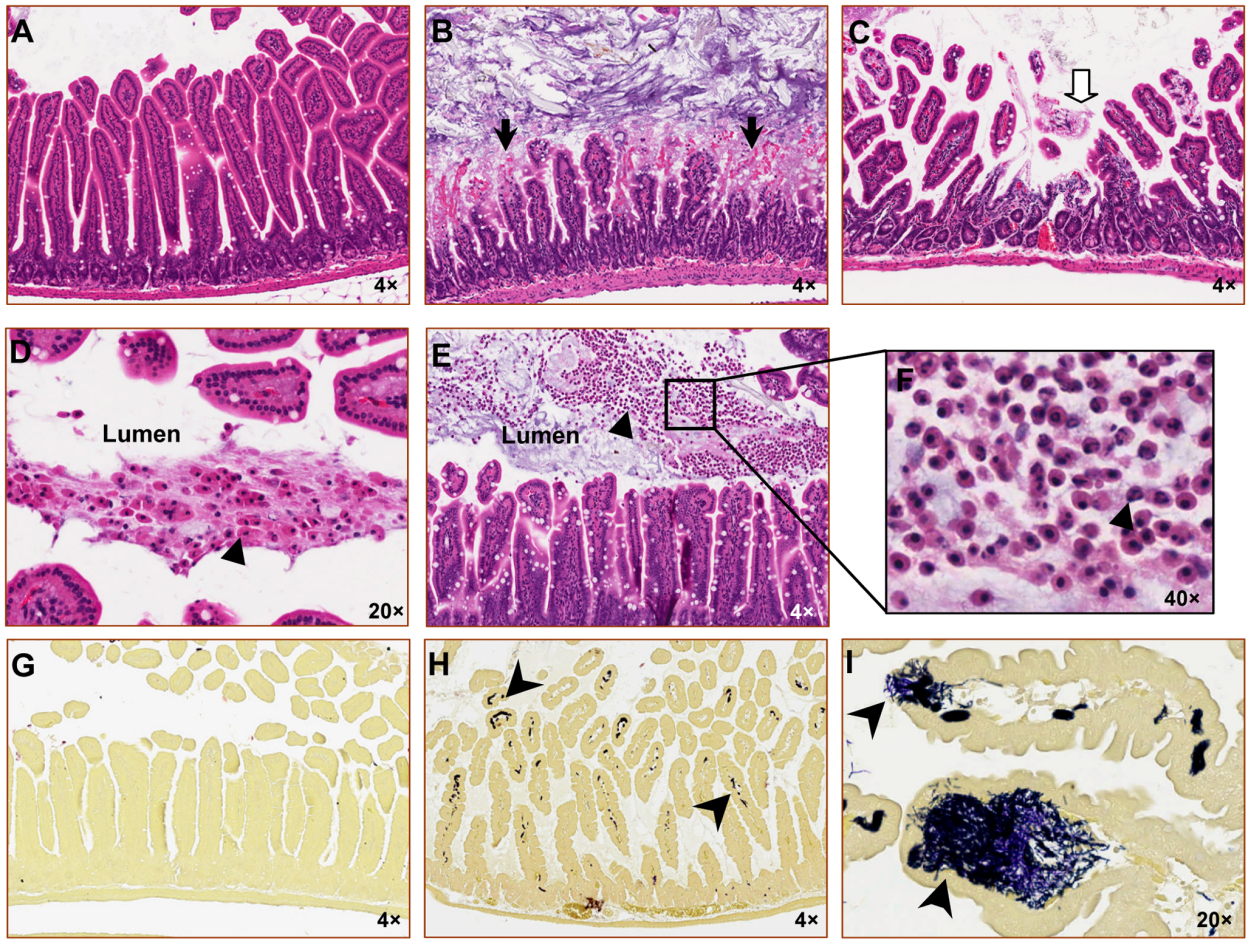
Murine gastrointestinal anthrax infection causes intestinal villus blunting and ulceration. Intestinal sections were obtained from moribund mice that had been infected with BaS or the vehicle control by gavage. Representative images were obtained at 4×, 20× or 40× magnifications as indicated (**A–F**). Representative H&E-stained sections from a control animal (**A**) or moribund BaS-treated animals (**B–F**) are shown. Black arrows indicate regions of epithelial destruction and bleeding (**B**). The block arrow points to a region of ulceration (**C**). Triangles identify sloughed epithelial cells (**D**) and/or inflammatory cell infiltration (**E**) found in the intestinal lumen. Image **F** shows a region within image **E** that was acquired at a higher magnification, revealing the presence of polymorphonuclear leukocytes. (**G–I**) Brown and Brenn-stained sections were assessed for evidence of anthrax infection. The section shown in **G** was obtained from a control non-infected mouse, whereas sections shown in **H** and **I** were from moribund mice. Gram-positive bacteria stain blue. Arrowheads identify clusters of Gram^+^ rods within the intestinal villi.

Compared to normal findings in vehicle-treated control animals (Figure 4A), histological evaluation of intestinal tissues from moribund animals with infection revealed villus blunting and ulceration, along with fragmentation and disruption of the normal villus structures (Figures 4B and 4C). In addition, the intestinal lumens of infected mice were marked by areas of sloughed epithelial cells, with scattered areas of intraluminal neutrophils (Figures 4D–F). Brown and Brenn staining of intestinal sections demonstrated that infected animals were characterized by invasion of the villi with Gram^+^ rods (Figures 4H and 4I), a feature not observed in control animals (Figure 4G). No evidence of bacteria was observed in the Peyer’s patches of infected mice (data not shown).

### Gastrointestinal Anthrax Infection in Mice is not Associated with Immune Activation

Other than scattered areas of neutrophils observed in the intestinal lumen (Figure 4F), there was little evidence that intestinal anthrax infection was marked by inflammatory immune responses. Reductions in neutrophils and T cell populations were noted in the small intestines of infected animals; B cell populations trended lower, but the change was not statistically significant (Figure S1A). The levels of these cell populations in colonic tissues were either reduced (B cells) or unchanged (T cells and neutrophils) compared to untreated control animals (Figure S1B). Moreover, intestinal anthrax infection in mice did not lead to activation of CD4^+^ and CD8^+^ T cells (i.e., CD44^+^CD62L^−^ and/or CD69^+^, Figures S2A and S2B), B220^+^ B cells (MHCII^+^ or CD40^+^, Figures S2C and S2D) or CD11c^+^ dendritic cells (MHCII^+^, CD80^+^, and/or CD86^+^, Figures S2E and S2F) obtained from mesenteric lymph node cells 48 h following gavage of *Bacillus anthracis* compared to those obtained from PBS-treated animals. Corresponding with these results, there was no significant increase pro-inflammatory cytokine production (MCP-1/CCL2, IL-1β, IL-6, TNF-α, and IFN-γ) in cultured tissue samples from the jejunum or ileum of infected mice (Figure S3), Moreover, there was no observed increase in mRNA levels of pro-inflammatory markers in jejunum tissue cultures obtained from mice 48 h following gavage with *Bacillus anthracis* compared to control mice (IL-6, IL-1β, CXCL2, and Nos2, Figure S4). Taken together, these data indicate suppression of immune responses during infection.

### Infection via the Gastrointestinal Route Leads to Widespread Dissemination

**Figure 5:**
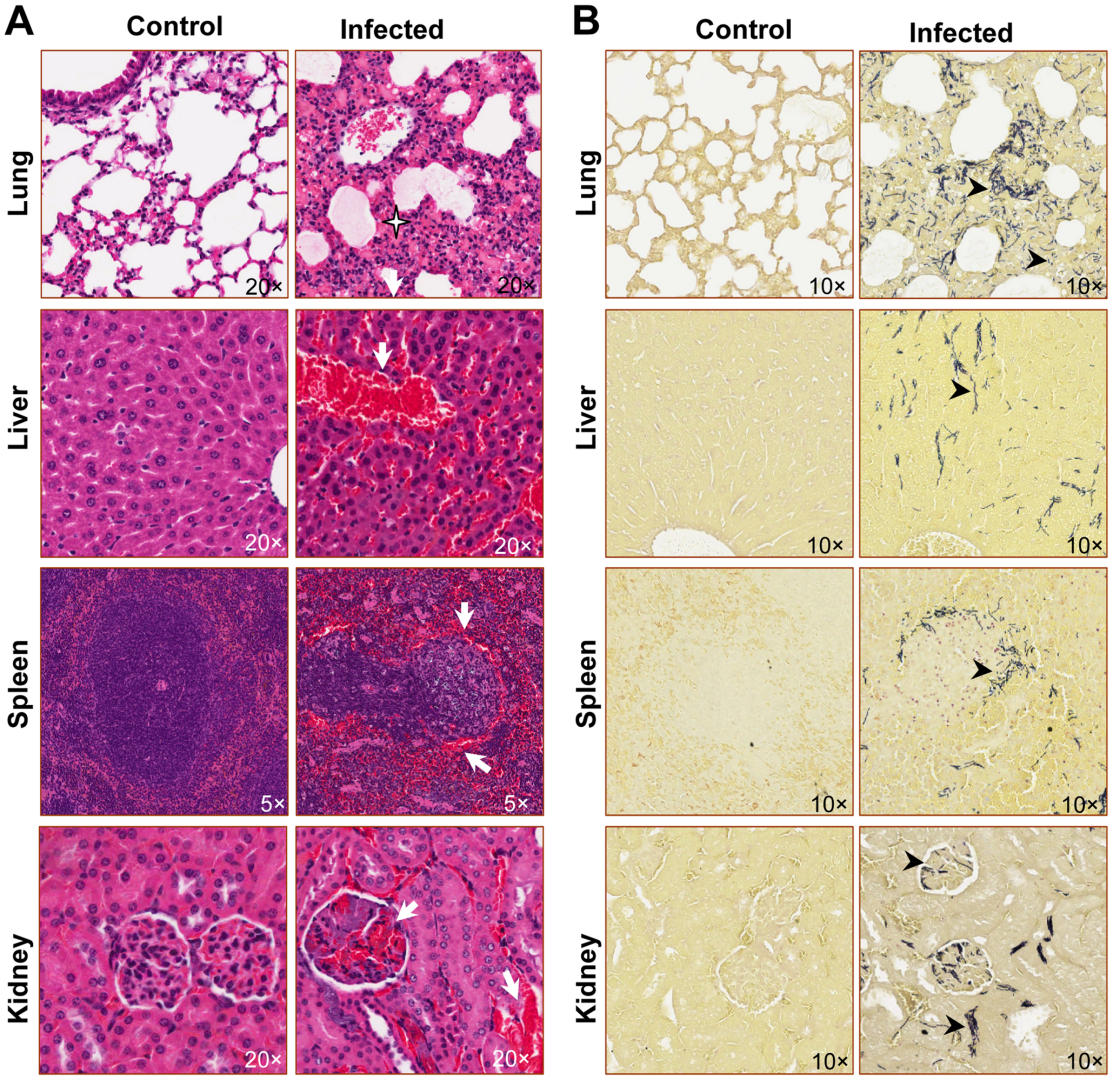
Murine gastrointestinal anthrax progresses to multi-organ infection. Tissue sections from multiple organs were collected from control or moribund GI-infected mice, and stained with H&E (**A**). The left columns show representative sections from untreated control mice, whereas columns on the right show representative sections from moribund infected mice. The star highlights proteinaceous material in the alveolar spaces, accompanied by alveolar destruction (lung section); white arrows indicate areas of hemorrhage and/or destruction of the normal tissue architecture (liver, spleen and kidney sections). Shown in **B** are sections stained with Brown and Brenn. Arrowheads indicate Gram+ rods, which were present throughout the interstitial areas of the tissues (lung, liver, spleen, and kidney) of infected animals (right column), but not in uninfected control animals (left column).

Tissues from major organs were subsequently assessed for signs of pathology and infection. Lungs from infected mice showed intra-alveolar hemorrhage, edema, and interstitial infiltrates (**Figure 5A**), associated with the presence of numerous Gram^+^ rods (**Figure 5B**). Liver sections revealed areas of hemorrhage and bacterial invasion (**Figures 5A and 5B**). Widespread damage was also observed in the spleens of infected animals, characterized by destruction of the normal architecture of the white pulp and accompanied by accumulation of Gram^+^ bacteria in the marginal zone (**Figures 5A and 5B**). In addition, the kidneys of infected mice were marked by infection, with large numbers of bacteria present in the renal glomeruli and interstitial areas (**Figure 5B**). Collectively, histopathology in moribund mice was characterized by hemorrhage, edema, and vascular congestion in a wide array of tissues, accompanied by marked white pulp depletion in the spleen. These pathological changes were similar to the observations in mice with disseminated infection acquired through inhalational or s.c. injection routes [12], [19], [20].

### Villus Blunting and Ulceration Occur Prior to Dissemination

**Figure 6:**
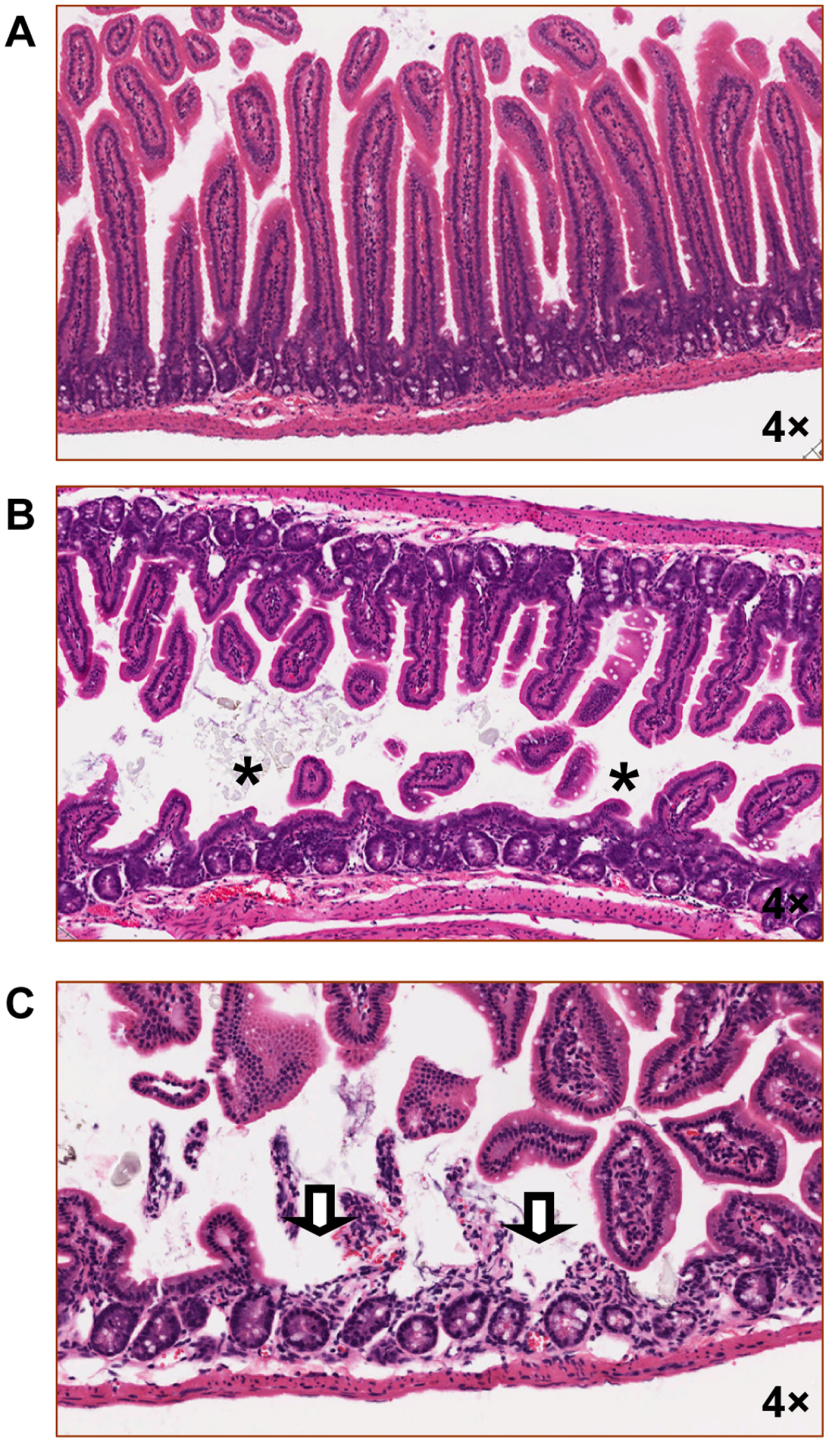
Gastrointestinal anthrax is marked by histological damage prior to hematological dissemination . Tissues were collected from mice 2 h (A) or 40 h (B and C) following intragastric challenge with BaS (1.3×10^9^). At neither of these time points were these BaS-challenged mice bacteremic. Representative images generated from H&E-stained sections from mice in each of these cohorts are shown. Asterisks indicate regions of villous blunting, whereas block arrows indicate focal regions of ulceration.

We next investigated the effects of gastrointestinal anthrax infection in time-course experiments. Mice gavaged with a high dose of bacteria (10^9^) showed little evidence of intestinal damage during the first 12 h following administration (data not shown). However, evidence of intestinal damage (hemorrhage, villus blunting and ulceration) was detectable in a majority of mice as early as 24 h following gavage (six out of ten animals). Moreover, intestinal pathology was observed in an entire cohort of mice (6/6 animals) that displayed no evidence of dissemination 40 h following gavage (e.g., normal activity, blood culture negative, and negative Brown and Brenn staining of tissues). This pathology was characterized by villus blunting and ulceration (**Figures 6B and 6C**, respectively). The villus architecture in infected animals was disrupted compared to that of uninfected control animals (**Figure 6A**), with pathological features that were very similar to what we observed previously in anthrax LT-treated mice [4], [5]. Taken together, these data are consistent with the primary site of infection being the epithelium of the gastrointestinal tract.

In additional studies, we compared the effects of infection on various regions in the intestinal tract, including the jejunum, ileum and colon. As summarized in **Table 1**, mild damage to the jejunum and ileum was noted as early as 24 h post gavage. The pathological scores in these regions of the small intestine increased over time, and nearly all moribund animals had evidence of damage in both of these areas. In contrast, pathological effects in the colon were rare in the first 48 h post anthrax gavage, and moribund mice showed only mild to moderate evidence of ulceration. Taken together, these data indicate that gastrointestinal anthrax in this murine model preferentially affects the small intestine compared to the colon.

**Table 1:** Summary of pathological findings*.

Pathological lesions		−	+	++	+++	# of mice examined
**0 h**	Jejunum	7				7
	Ileum	7				7
	Colon	5				5
**24 h**	Jejunum	9	4	1		14
	Ileum	6	7	1		14
**40–48 h**	Jejunum	4	2			6
	Ileum		6			6
	Colon	4	1			5
**Moribund**	Jejunum	3	4	6	1	14
	Ileum	2	4	2	6	14
	Colon	2	8	1		11

* The pathological scores of the intestinal specimens were classified into four grades as follows: no lesions seen (−); mild villous blunting and/or isolated hemorrhages (+); multifocal areas of moderate mucosal ulceration, villus blunting, and/or hemorrhage (++); severe mucosal ulceration characterized by severe structural destruction and abundant bacterial colonies in contact or within submucosal tissues and/or severe hemorrhage (+++).

### Murine Gastrointestinal Infection Model has Utility in Assessing Therapeutics

**Figure 7:**
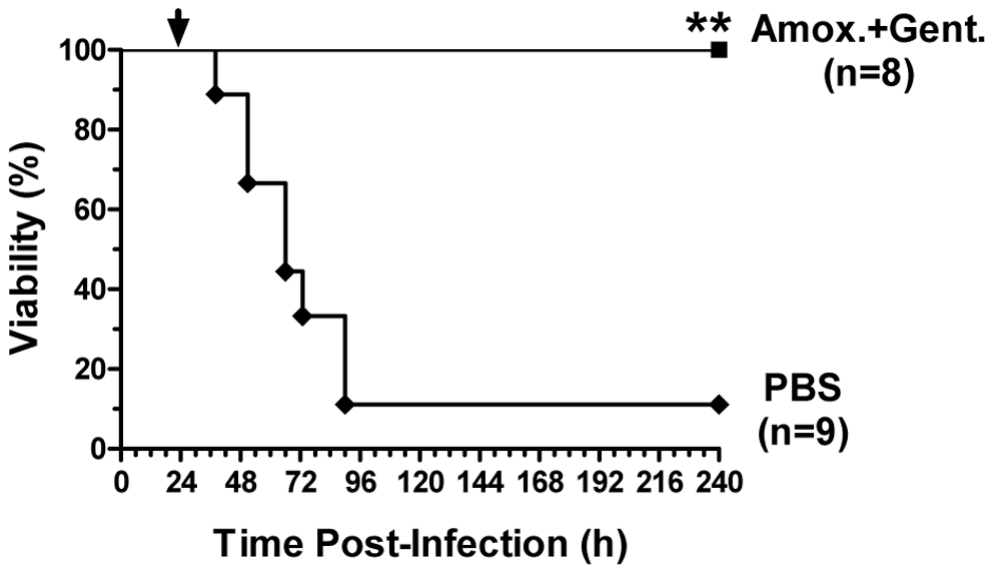
Delayed antibiotic treatment prevents lethality in murine gastrointestinal anthrax. A/J mice were infected via gavage with 1.2×10^9^ CFU of vegetative BaS. Twenty four hours later, the infected mice were treated with amoxicillin and gentamicin or PBS alone for 3 consecutive days. Mice were followed for 10 days post infection. **P<0.01. The *p* value represents the significance of the difference in survival between the antibiotic treatment and PBS control groups, calculated using the log-rank (Mantel-Cox) Test.

Having established a murine gastrointestinal anthrax model, we next investigated whether this model could be used to evaluate potential therapeutics. To this end, we investigated the efficacy of the combination of intraperitoneal amoxicillin and gentamicin in preventing disseminated infection and death. We used the identical dose regimen (16 mg/kg subcutaneous gentamicin per day and 100 mg/kg subcutaneous amoxicillin three times per day) that we used previously to prevent enteric bacterial infections following treatment with anthrax LT [4]. Although all 8/9 mice that received bacterial gavage alone died with disseminated infection experiment, all of the mice that received the antibiotic combination survived (**Figure 7**). This proof-of-concept experiment demonstrates the utility of this murine model in investigating the efficacy of potential anthrax therapeutics.

## Discussion

In the natural setting, anthrax infection is generally a disease of herbivores, which develop gastrointestinal infection while feeding in spore-infected fields [2], [3]. Presumably, *Bacillus anthracis* has evolved pathogenic mechanisms to facilitate a gastroenteric route of entry, yet the lack of informative animal models that mimic natural gastrointestinal anthrax has been a hurdle for investigating these mechanisms directly and comprehensively. The murine model that we have established will be a useful tool in overcoming this barrier.

We hypothesized that the exposure of gastrointestinal tissues to vegetative bacteria would better parallel the situation in ruminants following digestive fermentation, which, in turn, would lead to improved efficiency in establishing infection. Supporting this hypothesis, our dose-response experiments reveal that the LD_50_ for vegetative bacteria via this route of entry is approximately 2.3×10^7^, and an infectious dose as low as 2.3×10^6^ can lead to lethal infection. These results contrast those involving animal models infected with toxigenic *Bacillus anthracis* spores via the gastrointestinal tract, where a dose of 10^8^ could not establish infection in guinea pigs, rabbits or non-human primates [13], [14], [15]. Some reduction of the LD_50_ for mice has been reported when the spores are embedded in a thiabendazole paste prior to intragastric administration, but whether this manipulation mimics natural infection is unclear. Moreover, it is clear that consumption of infected meat from animals leads to infection in carnivores. This natural mode of transmission almost certainly involves exposure to vegetative bacteria growing in fresh meat, which contrasts infection acquired by ingesting infectious spores, which might occur in other settings (e.g., bioterrorism). Whether humans acquire gastrointestinal anthrax primarily through spores or vegetative bacteria would depend upon local customs (i.e., whether raw animal products or undercooked animal products are consumed).

Interestingly, increased infectivity of vegetative bacteria vs. spores was also observed in A/J mice challenged with BaS through subcutaneous injection. Whereas the LD_50_ of BaS spores via subcutaneous injection is 1.1×10^3^ [7], the LD_50_ is even lower than 10^2^ if vegetative bacteria are administered [19]. We speculate that factors produced by vegetative BaS may contribute to the early pathogenesis of anthrax infection acquired subcutaneously and that these pathogenic factors might be shared with gastrointestinal infection as well. In this regard, vegetative bacteria produce anthrax LT, which likely promotes infectivity. We previously demonstrated that LT-treated animals develop a compromise in the integrity of the intestinal barrier, marked by intestinal hemorrhage and ulceration [4], [5]. This breakdown is associated with development of systemic infections with commensal enteric bacteria [4]. Natural history experiments reveal that the pathology observed in murine gastrointestinal anthrax is very similar to that observed in anthrax LT-treated animals. Our findings are consistent with a model in which LT-mediated breakdown of the intestinal barrier leads to a portal of entry for enteric bacteria, including *Bacillus anthracis*. Interestingly, nearly 1/3 of animals that developed systemic infection with *Bacillus anthracis* were co-infected with other enteric organisms, similar to the systemic infections that we reported in anthrax LT-treated mice [4]. This murine infection model will serve as a useful tool in investigating the role of anthrax LT directly through comparison of gastrointestinal BaS infection vs. infection with an LT-deficient BaS strain.

It should be noted that the BaS strain is deficient in the capsule virulence factor of wild-type *Bacillus anthracis*. The mice used in the study were complement-deficient A/J mice, which are susceptible to BaS. For this reason, the model would not be amenable to assessing the role of the capsule virulence factor during gastrointestinal infection. Nevertheless, an animal model that involves the Sterne strain and small rodents has practical advantages with regard to space and biohazard considerations. Moreover, this represents the first practical model that can directly assess the role of anthrax LT in an infection acquired through administration of vegetative *Bacillus anthracis*.

Our findings are consistent with a model in which *Bacillus anthracis* infection leads to intestinal ulceration, likely through the action of anthrax LT [4], [5]. This breakdown in the intestinal barrier allows a portal of entry for dissemination. Interestingly, we observed no evidence of amplification of infection in the Peyer’s patches, which contrasted previous results derived from a murine model of gastrointestinal anthrax that utilized non-toxigenic spores [16], but is consistent with a model that involved spores imbedded in a thiobendazole paste [17]. Moreover, infection in our model did not lead to immune activation, likely due to the well-known immunosuppressive effects of anthrax LT. Infection with vegetative bacteria in our model leads to a rapid progression to hematological dissemination, which would be predicted to result from the observed destruction of the structural integrity of the musosal barrier and concomitant suppression of host immune defenses.

Importantly, we provide data from a proof-of-concept experiment demonstrating that this murine gastrointestinal infection has utility in assessing therapeutic efficacy *in vivo*. As the licensing of potential anthrax therapeutics will likely depend on efficacy data from animal models, the development of new animal models is of importance. This toxigenic murine model of gastrointestinal anthrax could fill an important niche in the tools available to assess new therapies for anthrax, especially those that target anthrax ET and/or LT.

## Materials and Methods

### Ethics Statement

Animal experiments were performed in accordance with animal protocol #WO2011-16, which was approved by the United States Food and Drug Administration Center for Biologics Evaluation and Research (CBER) Institutional Animal Care and Use Committee, in accordance with the U.S. Public Health Service Policy on Humane Care and Use of Laboratory Animals (Assurance # A4295-01). The CBER animal program is accredited by Association for Assessment and Accreditation of Laboratory Animal Care International. Animal welfare was assessed at least twice per day.

### Mice, Bacterial Strain and Gastrointestinal Challenge Experiments

Female A/J mice used in the experiments were obtained from The Jackson Laboratory (Bar Harbor, ME, USA) and were 9–12 weeks of age at the time of the experiments. Mice were allowed at least one week to acclimatize to the animal facilities prior to experimentation. Mice were housed using standard cages that had a capacity for up to 5 animals. Food and water were provided *ad libitum*, with the exception of the fasting period described below. Study animals that became moribund were euthanized. Mice were euthanized either via terminal exanguination under anesthesia [i.p. administration of ketamine (60–70 mg/kg) and xylazine (12–14 mg/kg)], carbon dioxide inhalation in a euthanasia chamber, or by cervical dislocation by experienced animal handlers (in cases requiring rapid tissue collection).

*Bacillus anthracis* (Sterne strain 7702; BaS) was kindly provided by Dr. Tod Merkel [6]. All experiments with this strain were carried out using biosafety level 2 procedures. To prepare vegetative BaS bacteria, 50 µL of frozen stock was incubated in 5 mL BBL Brain Heart Infusion medium (BHI; Becton, Dickinson and Company, Franklin Lakes, NJ, BD 221813) at 37°C for 16 h (overnight). The overnight culture was diluted 1∶1 with fresh, pre-warmed BHI medium and cultured an additional 6 h, at which time the density was at least 2×10^8^. This estimate was based on repeated experiments with similarly prepared suspensions. However, the actual challenge dose (colony forming units, CFU) was calculated post-hoc via dilution and plating on BHI plates. To distinguish between bacilli and spores in culture, a fraction of the bacterial culture was heat-treated at 60°C for 30 min to kill vegetative bacilli. Heat-treated and untreated samples were serially diluted and plated, and results were recorded as numbers of CFU. Using the culture conditions described above, we determined that the concentration of heat-resistant colony forming units (spores)/vegetative bacteria was 4/10^8^.

### Gastrointestinal Infection Experiments

For gastrointestinal challenge experiments, the mice were first fasted for 16 hours to allow the passage of stomach contents. Mice were then received 60–70 mg/kg intraperitoneal ketamine and 12–14 mg/kg xylazine for anesthesia. When the mice were deeply sedated, 50 µl of 8.5% (W/V) NaHCO_3_ was administrated, which was followed immediately by 150 µl of the bacteria suspension that had been concentrated (4000 g×8 min) or diluted in varying amounts of culture medium (pre-warmed to 37°C) to provide the desired concentration for administration. As a control to assess a potential role for bacterial spores, some mice were gavaged with heat-treated bacteria (60°C for 30 min). Single-use plastic feeding tubes (20 GA 38 mm, Instech, Plymouth Meeting, PA) were used for intragastric administration. After the procedure, mice were returned to barrier housing and received food and water *ad libitum*. To eliminate allocation bias in time-course experiments, mice were pre-segregated into treatment groups corresponding to each time point to be assessed. A clinical assessment of the mice was performed by monitoring body weight, rectal temperature, locomotor activity and general appearance [21]. As described in the figure legend, these parameters were scored and added together to provide a combined clinical score.

### Flow Cytometry

Fluorescent dye–labeled antibodies to the cell surface markers CD4 (RM4-5), CD8 (53–6.7), TCRβ (H57-597), CD69 (H1.2F3), CD62L (MEL-14), CD44 (1M7), B220 (RA3-6B2), MHCII (M5/114.15.2), CD40 (1C10), CD11c (N418), CD80 (16-10A1) and CD86 (2F4) were purchased from eBiosciences (San Diego, CA), along with corresponding isotype control antibodies. Mesenteric lymph nodes were dissected from euthanized mice and were digested in collagenase (2 mg/mL; Sigma, St. Louis, MO) dissolved in DMEM medium (Invitrogen, Grand Island, NY) containing 5% FBS at 37° for 45 min. After digestion, the cells were washed twice and resuspended in DMEM medium. Mesenteric lymph node cells were incubated for 15 min on ice with these specific antibodies or corresponding isotype control antibodies using standard methods [22]. All samples were acquired and analyzed with an LSR II (Becton Dickinson, Franklin Lakes, NJ) and FlowJo software (TreeStar).

### Cytokine Measurements

The assay to detect cytokine levels in supernatants of cultured tissues was adopted from a published method [23]. In brief, longitudinally-cut intestinal biopsies were washed once with PBS and three times in RPMI 1640 (with antibiotics, pre-warmed to 37°C; Hyclone Laboratory, Inc., Logan, Utah). Five 1 cm sections of tissue were then placed in 24-well flat-bottom culture plates containing 1 mL of serum-free RPMI 1640 medium supplemented with penicillin (100 µg/ml) and streptomycin (100 µg/ml) and maintained at 37°C. Following culture for 18 h, culture supernatants were collected and tested for cytokine concentrations. Cytokine assays were performed in duplicate using a customer-designed Procarta multiplex bead-based kit that assessed MCP-1, IL-1β, IL-6, TNF-α, and IFN-γ (Affymetrix, CA). Bead fluorescence was measured and cytokine levels analyzed using a BioPlex 200 analyzer (Luminex; Bio-Rad) and BioPlex Manager software (Bio-Rad).

### Tissue Section Immunofluorescence Staining

Paraffin embedded tissue sections (5 µm) were prepared from intestine tissues, and sections were de-paraffinized in xylene and rehydrated in a series of graded alcohols. Antigen retrieval was then performed in Antigen Unmasking Solution (H-3300, Vector Burlingame, CA). Slides were incubated at 4°C overnight at a 1∶150 dilution with the following primary antibodies: CD3 (NBP1-72167, Novus, Littleton, CO), B220 (RA3-6B2, eBioscience, San Diego, CA) or Gr-1 (RB6-8C5, eBioscience, San Diego, CA). Following three 10 min washes in PBS, slides were incubated with Alexa Fluor-labeled secondary antibody (1∶500) at room temperature for 90 min. The stained sections were mounted in Prolong Gold Anti-fade reagent (P36935, Invitrogen) and examined using a Keyence BZ-9000 All-in-one fluorescence microscope.

### Quantitative Reverse Transcription-PCR

RNA was extracted from frozen tissues using a Mini RNeasy Kit (Qiagen, Gaithersburg, MD) and treated with DNase (DNA-free, Ambion, Austin, TX) to remove DNA contamination. Reverse transcription (RT) was performed using the SuperScript Vilo cDNA Synthesis kit (11754-050, Invitrogen, Grand Island, NY). Gene expression levels were measured by quantitative RT-PCR using the ABI Prism 7900 Real-time PCR system (Applied Biosystems, Foster City, CA). PCR reactions (20 µl total volume) included cDNA, 100 nM primers and 10 ul of SYBR Green MasterMix (Applied Biosystems, Foster City, CA). To obtain the relative quantification of the mRNA of the genes, the mRNA levels of the genes were normalized to β-actin mRNA levels in each sample, which were determined simultaneously by the same method. The following primer pairs were used for RT-PCR: CXCL2, Forward 5′-AACATCCAGAGCTTGAGTGTGA-3′ and Reverse 5′-TTCAGGGTCAAGGCAAACTT-3′;

IL-6, Forward 5′-ATGGATGCTACCAAACTGGAT-3′ and Reverse 5′-TGAAGGACTCTGGCTTTGTCT-3′; IL-1β, Forward 5′-CAACCAACAAGTGATATTCTCCATG-3′and Reverse 5′- GATCCACACTCTCCAGCTGCA-3′; iNOS2, Forward 5′-CAGCTGGGCTGTACAAACCTT-3′and Reverse 5′- CATTGGAAGTGAAGCGTTTCG; and β-actin, Forward 5′-TTCCATCATGAAGTGTGACGTT-3′ and Reverse 5′ –CTCAGGAGGAGCAATGATCTTG-3′.

### Antibiotic Efficacy Experiments

Female A/J mice were infected via gavage with 1.2×10^9^ CFU of vegetative BaS. Twenty four hours later, the infected mice were treated with amoxicillin and gentamicin or PBS alone for 3 consecutive days. The following antibiotic regimen was used: 16 mg/kg subcutaneous gentamicin per day and 100 mg/kg subcutaneous amoxicillin three times per day. Mice were monitored at least every 6 h until the termination of the experiment (10 days post infection). Blood culture results from all animals that died showed disseminated anthrax infection.

### Histological Assessments

Mice were euthanized when moribund or at specific time points post-infection as indicated in the figure legends. The intestines and other organs were dissected and fixed in neutral buffered formalin. Paraffin sections were prepared and stained with hematoxylin and eosin (H&E) and/or Brown and Brenn (B&B) by Histoserv, Inc (Germantown, MD). The stained slides were scanned using an Aperio ScanScope (ScanScope, Aperio, CA) and acquired using 20×or 40×magnification. These digital images were converted into TIF files for generating figures. The magnification factors are provided within the figures or figure legends. Histological assessments and scoring were made by an investigator blinded to the treatment cohort from which the sections were obtained.

### Bacterial Isolation and Identification

Bacterial isolates were cultured on blood agar plates (RO1202, Remel, Lenexa, KS) using blood samples obtained aseptically from cardiac puncture. In some cases, blood samples were also cultured on BHI plates (#221569, Becton Dickinson and Company). Bacterial identifications were performed using the Omnilog Microbial Identification System (Biolog Inc., Hayward, CA). Bacterial colonies were isolated and suspended in a nutrient-deficient inoculating medium containing tetrazolium redox dyes provided by Biolog Inc., using the manufacturer’s recommended protocol. Suspended bacteria were then incubated in 96 well MicroPlates provided by Biolog Inc., which were then incubated in the identification instrument at 33°C for analysis. Identifications were made through colorimetric analysis of the plates, which produced a metabolic fingerprint that could be compared with a software database that included a fingerprint for BaS.

### Statistical Methods

Statistical analyses were performed using GraphPad Prism 5 software. For survival studies, the log-rank (Mantel-Cox) test was used.

## Supporting Information

Figure S1 **Intestinal anthrax infection is not associated with inflammatory cell infiltrates in intestinal tissues.** Intestinal tissue sections were prepared from moribund, infected mice and uninfected control mice. The average numbers of T cells (positive for CD3), B cells (positive for B220) and neutrophils (positive for Gr-1) in small intestinal (jejunum) (**A**) and colonic (**B**) sections were generated from six to twelve randomly picked microscopic fields per animal (20×, Keyence BZ-9000 fluorescence microscope). The numbers of analyzed animals are provided in parentheses on the X-axes. Average values for each cohort are shown as horizontal bars; statistical significance (p-value) was determined using the Student’s t-test.
(TIF)

Figure S2 ***Bacillus anthracis*** **infection does not activate T cells, B cell and dendritic cells.** Mesenteric lymph node cells were obtained from mice 48 h following gavage of PBS (control, C) or BaS (infected, I), stained with relevant antibodies, and assessed by flow cytometry. (**A**) T cells were identified through TCR-β staining (left panels); TCR-β^+^-gated cells were subsequently assessed for CD4 and CD8 expression (right panels). (**B**) CD4^+^ and CD8^+^ subsets, in turn, were assessed for CD44, CD62L and CD69 expression. Activated T cells are CD44^+^CD62L^−^ and/or CD69^+^. (**C**) B cells were identified through B220 staining (left panels). (**D**) B220^+^-gated cells were subsequently assessed for MHCII and CD40, markers of B cell activation. (**E**) Dendritic cells were identified through CD11c staining. (**F**) CD11c^+^-gated cells were subsequently assessed for MHCII, CD80, and CD86 expression, markers of dendritic cell activation.
(TIF)

Figure S3 **Intestinal anthrax infection has minimal effects on inflammatory cytokine production.** Samples from the jejunum and ileum were obtained 48 h following gavage with BaS (infected, I) or PBS (control, C) and were cultured *ex vivo*. Cytokine/chemokine levels in the supernatants of these *ex vivo* small intestine cultures (normalized to tissue weight) were assessed and are shown, with each dot representing the results for one animal (n = 4 for each treatment group).
(TIF)

Figure S4 **Intestinal anthrax infection does not increase inflammatory gene expression.** Using RT-PCR, mRNA levels of the indicated pro-inflammatory genes were measured in jejunum samples obtained from mice 48 h following gavage with PBS (control, open bar,) or BaS (infected, closed bar). mRNA levels were first normalized to β-actin levels. Mean levels of each cytokine in PBS controls were arbitrarily assigned a relative level of 1 (n = 5/group; SEM values are shown).
(TIF)
