# Supplementary figures and images for: A New Murine Model for Gastrointestinal Anthrax Infection
Source: PLoS One. 2013 Jun 18;8(6):e66943. doi: 10.1371/journal.pone.0066943 (PMC3688947; doi:10.1371/journal.pone.0066943)

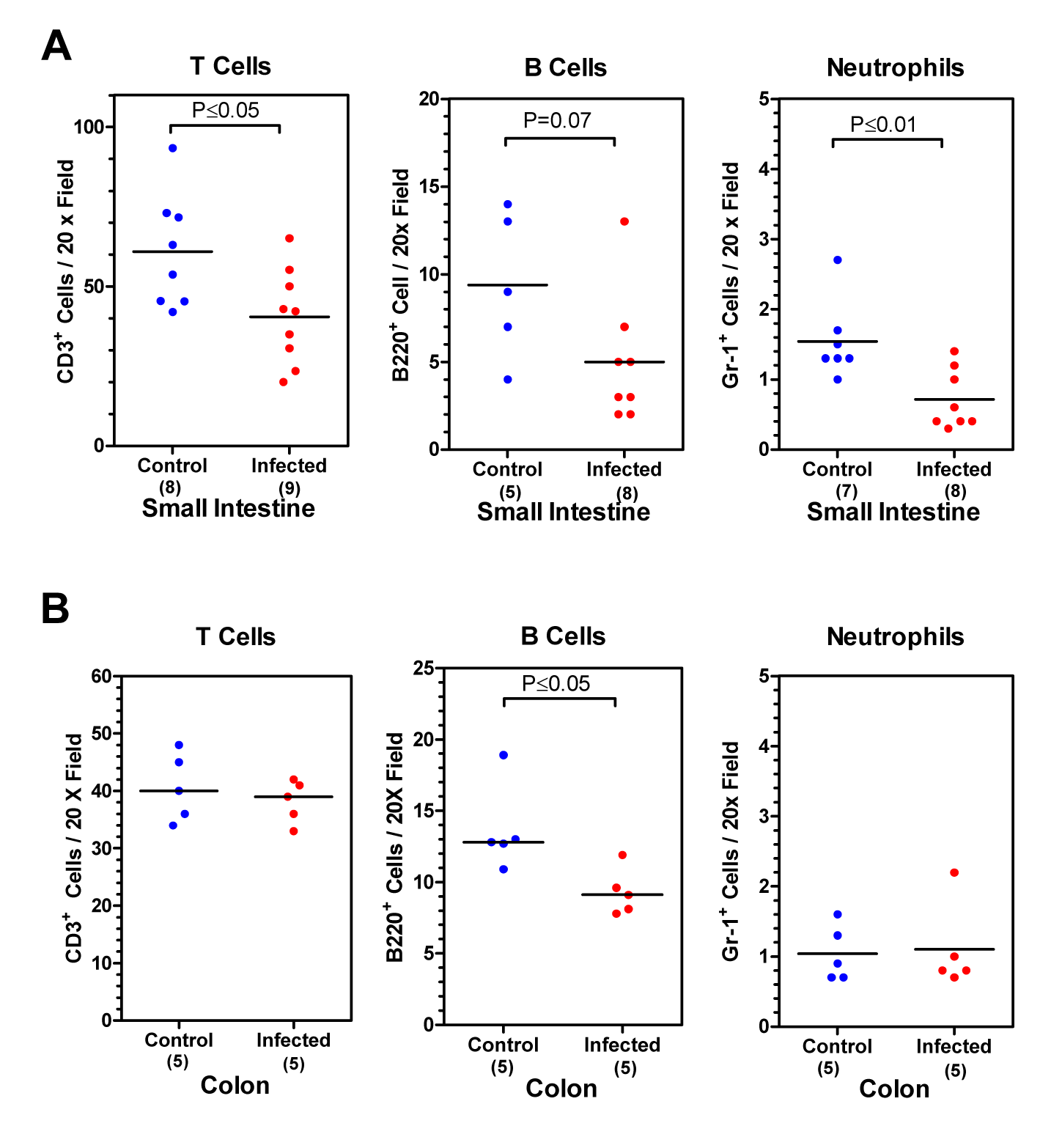

Supplement: Figure S1 — Intestinal anthrax infection is not associated with inflammatory cell infiltrates in intestinal tissues. Intestinal tissue sections were prepared from moribund, infected mice and uninfected control mice. The average numbers of T cells (positive for CD3), B cells (positive for B220) and neutrophils (positive for Gr-1) in small intestinal (jejunum) (A) and colonic (B) sections were generated from six to twelve randomly picked microscopic fields per animal (20×, Keyence BZ-9000 fluorescence microscope). The numbers of analyzed animals are provided in parentheses on the X-axes. Average values for each cohort are shown as horizontal bars; statistical significance (p-value) was determined using the Student’s t-test. (TIF) [file pone.0066943.s001.tif]

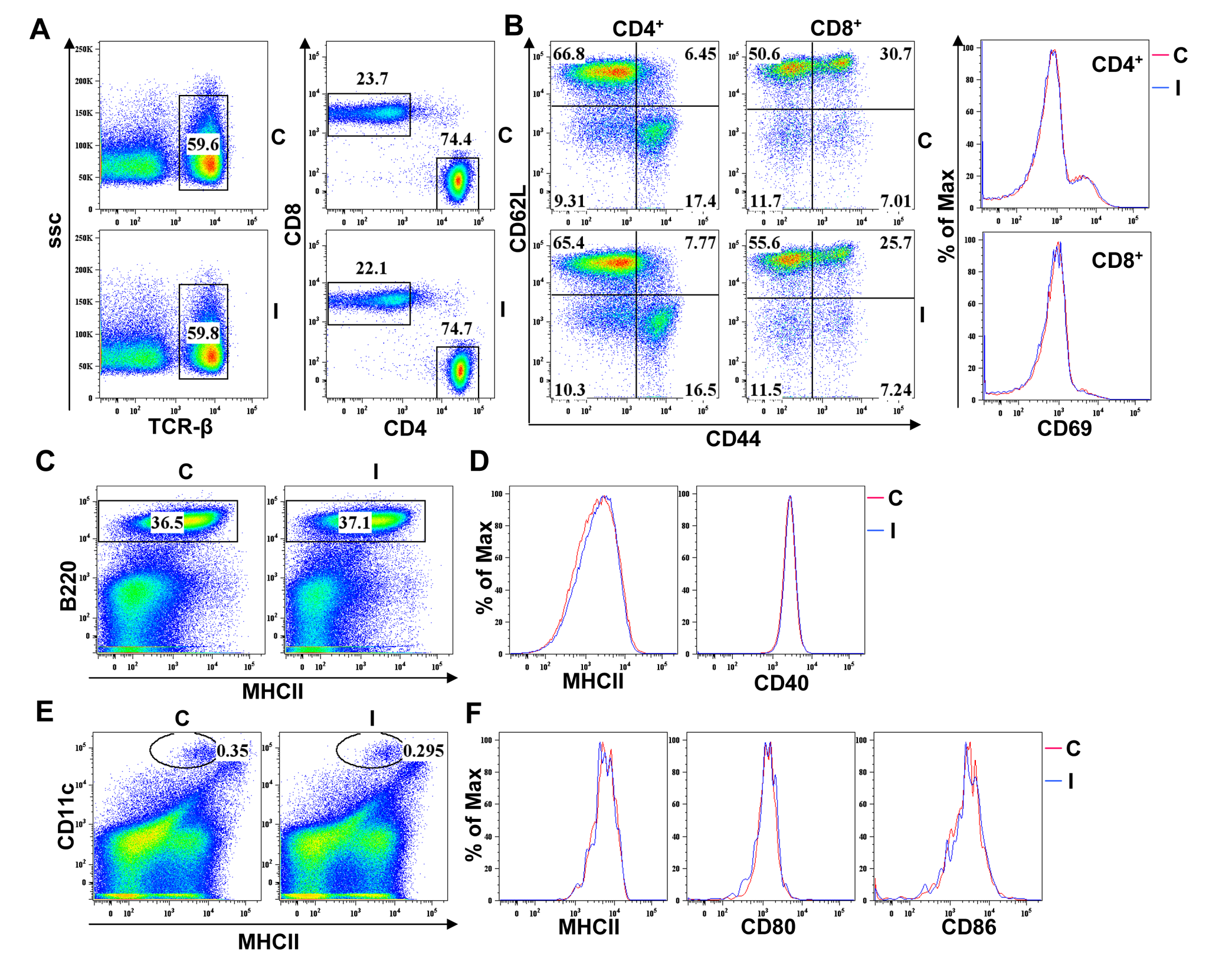

Supplement: Figure S2 — Bacillus anthracis infection does not activate T cells, B cell and dendritic cells. Mesenteric lymph node cells were obtained from mice 48 h following gavage of PBS (control, C) or BaS (infected, I), stained with relevant antibodies, and assessed by flow cytometry. (A) T cells were identified through TCR-β staining (left panels); TCR-β+-gated cells were subsequently assessed for CD4 and CD8 expression (right panels). (B) CD4+ and CD8+ subsets, in turn, were assessed for CD44, CD62L and CD69 expression. Activated T cells are CD44+CD62L− and/or CD69+. (C) B cells were identified through B220 staining (left panels). (D) B220+-gated cells were subsequently assessed for MHCII and CD40, markers of B cell activation. (E) Dendritic cells were identified through CD11c staining. (F) CD11c+-gated cells were subsequently assessed for MHCII, CD80, and CD86 expression, markers of dendritic cell activation. (TIF) [file pone.0066943.s002.tif]

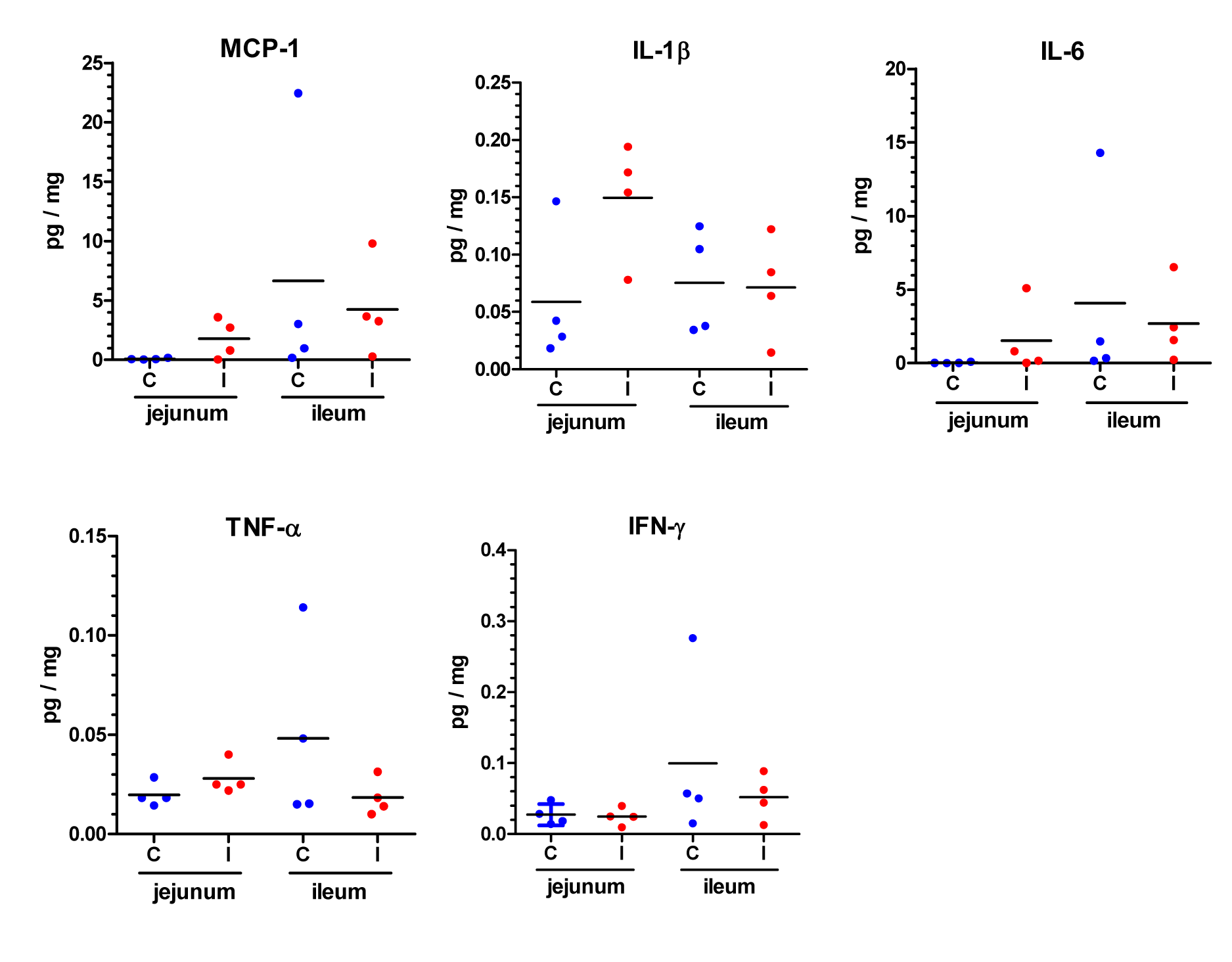

Supplement: Figure S3 — Intestinal anthrax infection has minimal effects on inflammatory cytokine production. Samples from the jejunum and ileum were obtained 48 h following gavage with BaS (infected, I) or PBS (control, C) and were cultured ex vivo. Cytokine/chemokine levels in the supernatants of these ex vivo small intestine cultures (normalized to tissue weight) were assessed and are shown, with each dot representing the results for one animal (n = 4 for each treatment group). (TIF) [file pone.0066943.s003.tif]

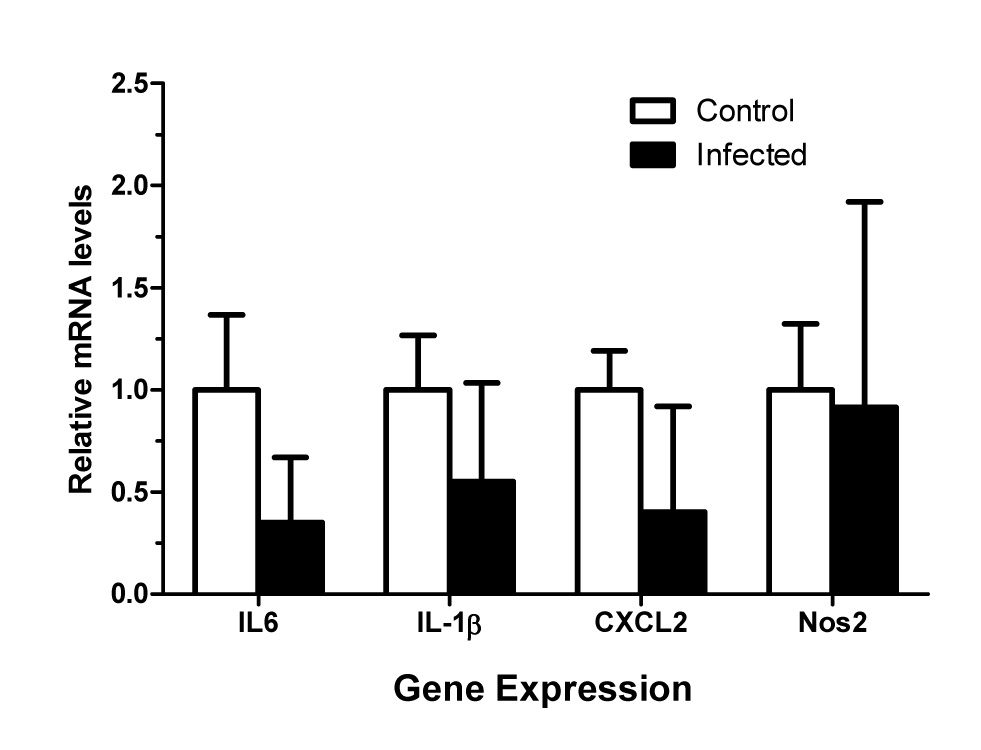

Supplement: Figure S4 — Intestinal anthrax infection does not increase inflammatory gene expression. Using RT-PCR, mRNA levels of the indicated pro-inflammatory genes were measured in jejunum samples obtained from mice 48 h following gavage with PBS (control, open bar,) or BaS (infected, closed bar). mRNA levels were first normalized to β-actin levels. Mean levels of each cytokine in PBS controls were arbitrarily assigned a relative level of 1 (n = 5/group; SEM values are shown). (TIF) [file pone.0066943.s004.tif]
